# Osteoarthritis and the risk of cardiovascular disease: a meta-analysis of observational studies

**DOI:** 10.1038/srep39672

**Published:** 2016-12-22

**Authors:** Haoran Wang, Jing Bai, Bing He, Xinrong Hu, Dongliang Liu

**Affiliations:** 1 Department of Cardiology, Luohe Central Hospital, The First Affiliated Hospital of Luohe Medical College, Luohe 462000, China; 2 Department of Endocrinology and Diabetes, Luohe Central Hospital, The First Affiliated Hospital of Luohe Medical College, Luohe 462000, China; 3 Department of Joint Surgery, Luohe Central Hospital, The First Affiliated Hospital of Luohe Medical College, Luohe 462000, China

## Abstract

Previous observational studies have suggested a potential relationship between osteoarthritis (OA) and the risk of cardiovascular disease (CVD), with conflicting results. We aimed to provide a systematic and quantitative summary of the association between OA and the risk of CVD. We searched Medline and EMBASE to retrieve prospective and retrospective studies that reported risk estimates of the association between OA status and CVD risk. Pooled estimates were calculated by a random effects model. The search yielded 15 articles including a total of 358,944 participants, including 80,911 OA patients and 29,213 CVD patients. Overall, the risk of CVD was significantly increased by 24% (RR: 1.24, 95% CI: 1.12 to 1.37, P < 0.001) in patients with OA compared with the general population, with no significant publication bias. Furthermore, sensitivity analysis indicated that our results were robust and were not influenced by any one study. In conclusion, this meta-analysis provides strong evidence that OA is a significant risk factor for CVD.

Cardiovascular disease (CVD), such as ischemic heart disease (IHD), congestive heart failure (CHF), transient ischemic attacks (TIA), and stroke, is a leading cause of morbidity and mortality in the general population worldwide. According to the World Health Organization, 17.5 million people die each year from CVD, constituting approximately 30% of all deaths worldwide^1^. Cardiovascular diseases therefore place a great burden on people, the economy, and society in general. However, cardiovascular disorders are largely preventable. The identification of new cardiovascular risk factors and interventions to modify these factors is of great importance for addressing the current epidemic.

Osteoarthritis (OA) is also a major cause of morbidity and healthcare expenditures and affects approximately 15% of the population^2^. By age 65, 80% of the population has radiographic evidence of OA, and 60% are experiencing symptoms of OA^3^.

Recent epidemiological studies have suggested a potential relationship between OA and CVD, with conflicting results. Whereas Jonsson *et al*. found a linear association between the severity of hand OA and atherosclerosis in the AGES Reykjavik study^4^, Haugen *et al*. concluded that symptomatic hand OA but not radiographic hand OA was associated with an increased risk of coronary heart disease events using the data from the Framingham Heart Study^5^. Moreover, the results from the Rotterdam Study indicated that disability, not OA, predicted cardiovascular disease^6^. This topic remains controversial, and the meta-analysis presented here provides a valid and up-to-date summary of the relevant literature. We aimed to determine whether OA patients are at increased risk of CVD. We also evaluated whether this association differs by type of OA or CVD.

## Results

### Literature search

**Figure 1:**
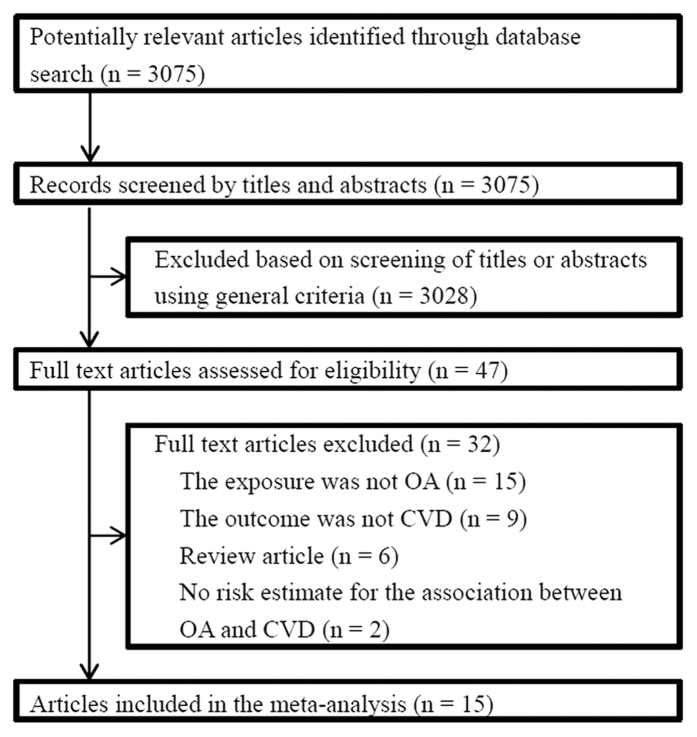
Literature Search for the Meta-analysis.

Overall, 3,075 references were initially identified. After the initial screening of titles and abstracts, a total of 3,028 articles were excluded, leaving 47 articles for retrieval. Full text assessment of these articles resulted in 15 eligible articles that met our inclusion criteria^4^,^5^,^6^,^7^,^8^,^9^,^10^,^11^,^12^,^13^,^14^,^15^,^16^,^17^,^18^. The total number of participants included was 358,944, with 80,911 OA patients and 29,213 CVD patients. Figure 1 displays the process of selection of studies.

### Study characteristics

Table 1 shows the characteristics of the 15 included articles. The articles included in our systematic review were quite heterogeneous. Five were retrospective studies^4^,^9^,^12^,^14^,^16^, and 10 were prospective studies^5^,^6^,^7^,^8^,^10^,^11^,^13^,^15^,^17^,^18^. The studies were conducted in the United States^5^,^7^,^10^,^14^, Europe^4^,^6^,^8^,^9^,^11^,^12^,^18^, Canada^15^,^16^, and Japan^17^. Thirteen studies included men and women^4^,^5^,^6^,^8^,^9^,^10^,^12^,^13^,^14^,^15^,^16^,^17^,^18^, and four reported results separately by sex^4^,^8^,^13^,^16^; the other two studies included only women^7^,^11^.

Features of exposure varied across studies. Although 12 studies ascertained OA by either radiographic results or medical records^4^,^5^,^6^,^7^,^8^,^9^,^11^,^12^,^13^,^17^,^18^, the other three used questionnaires to confirm OA status^10^,^14^,^16^. The studies focused on different sites of OA, including the hand^4^,^6^,^8^,^11^,^18^, knee^6^,^11^,^17^,^18^, or hip^6^,^7^,^18^.

The method of outcome ascertainment varied across articles. Although the majority of articles ascertained CVD by medical records, three articles used questionnaires to ascertain CVD^4^,^14^,^16^. Two articles^6^,^12^ reported an overall risk outcome, 3^5^,^14^,^18^ reported both an overall risk outcome and separately for different outcomes, 6^7^,^8^,^10^,^11^,^13^,^17^ reported risk estimate for one specific outcome. The others^4^,^9^,^15^,^16^ reported risk estimates separately for different outcomes; and in this case, the risk estimate for the most prevalent type of outcome serves as a surrogate for CVD risk estimate in the pooled analysis.

Although the included articles were quite heterogeneous, the inter-reviewer reliability for data extraction was almost perfect (kappa ranged 0.99–1.00). All studies were rated as medium to high quality.

### Systematic review of evidence

Fifteen articles provided 19 risk estimates of the association between OA and CVD risk. Of these 19 estimates, 11 reported that OA was associated with a significantly increased risk of CVD, five that OA was associated with a non-significantly increased CVD risk, and three that OA was associated with a non-significantly decreased CVD risk. No study included in this systematic review reported that OA was associated with a significantly decreased risk of CVD.

For the non-parametric sign test, we considered whether studies reported an increased or decreased risk of CVD associated with OA, regardless of significance level or magnitude of effect. Overall, 16 risk estimates found that OA was associated with an increased risk of CVD, and three found that OA was associated with a decreased risk of CVD. The sign test (P = 0.004) rejected the null hypothesis of equal CVD risk in patients with and without OA.

### Meta-analysis

**Figure 2:**
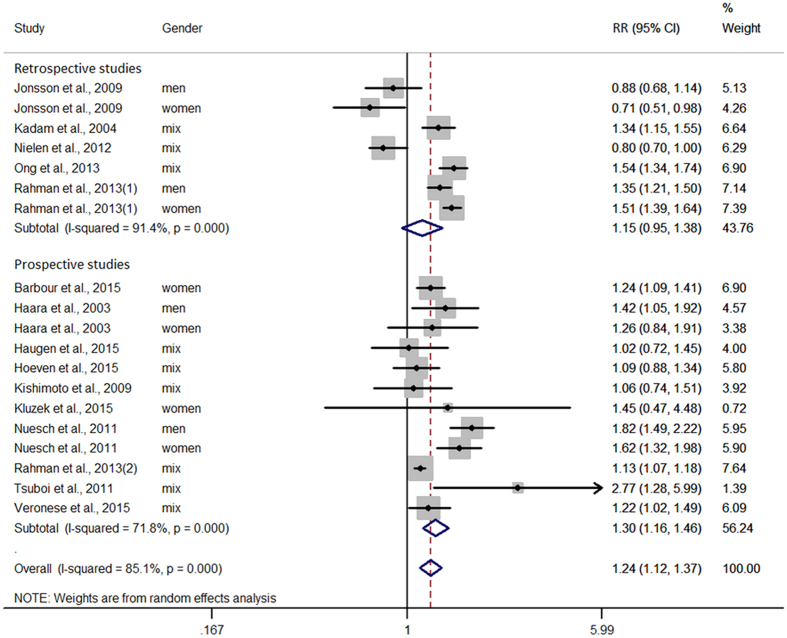
Association between OA and CVD risk. Estimates are derived from random effects. Dots indicate relative risks. Horizontal lines indicate 95% confidence intervals for relative risks. Diamonds represent pooled relative risk estimates with 95% confidence intervals.

Figure 2 displays the results of the meta-analysis of the 15 articles. A high level of between-study heterogeneity was detected, with *I*^2^ = 85.1%. Overall, the risk of CVD was significantly increased by 24% (RR: 1.24, 95% CI: 1.12 to 1.37, P < 0.001) in patients with OA compared with the general population. No significant publication bias was observed according to the Egger test (P = 0.659) or funnel plot (Supplementary Figure S1).

A sensitivity analysis was performed to evaluate the impact of additional study characteristics on the pooled RR. First, we calculated separate estimates by study design and found that the prospective studies (12 estimates) had a pooled RR of 1.30 (95% CI: 1.16 to 1.46, P < 0.001) and the retrospective studies (seven estimates) had a pooled RR of 1.15 (95% CI: 0.95 to 1.38, P = 0.147).

Second, we excluded studies that ascertained OA or CVD status by questionnaire. The remaining studies had a pooled RR of 1.26 (95% CI: 1.12 to 1.42, P < 0.001). Finally, we excluded individual study estimates one at a time to examine the influence of each study on the overall RR. We found that the omission of any one study did not appreciably change the pooled RR (Supplementary Figure S2).

### Subgroup analysis and meta-regression analysis

When subgroups were stratified by study design, we found a significant effect of prospective studies (RR: 1.30, 95% CI: 1.16 to 1.46, P < 0.001) and a non-significant effect of retrospective studies (RR: 1.15, 95% CI: 0.95 to 1.38, P = 0.147). The stratified analysis according to OA site included 3 subgroups, hand (RR: 1.03, 95% CI: 0.85 to 1.25, P = 0.749), knee (RR: 1.30, 95% CI: 1.00 to 1.69, P = 0.047) and hip (RR: 1.23, 95% CI: 1.11 to 1.38, P < 0.001). When studies were stratified according to confirmation methods for OA, we found a RR of 1.47 (95% CI: 0.91 to 2.39, P = 0.118) for clinical OA and a RR of 1.23 (95% CI: 1.02 to 1.48, P = 0.028) for radiographic OA. When studies were stratified according to different CVD types, we found significant results for the IHD (RR: 1.33, 95% CI: 1.20 to 1.46, P < 0.001), CHF (RR: 1.40, 95% CI: 1.13 to 1.73, P = 0.002) and cardiovascular death (RR: 1.53, 95% CI: 1.27 to 1.84, P < 0.001) subgroups, whereas a non-significant result was found for the stroke subgroup (RR: 1.11, 95% CI: 0.96 to 1.29, P = 0.16). Finally, we considered whether subgroup analysis reported a higher or lower CVD risk associated with OA, regardless of significance level. The results indicated that all subgroup estimates found that OA was associated with an increased risk of CVD. The results of the subgroup analyses are summarized in Table 2.

The impact of age and follow up time on the pooled result was explored by meta-regression analysis. As illustrated in Supplementary Figures S3, S4 and S5, there was no significant impact of age and follow up time on the pooled result.

## Discussion

This is the most comprehensive systematic review and meta-analysis of published observational studies assessing the relationship between OA and CVD risk. The results of our meta-analysis demonstrate that OA is associated with a significantly increased risk of CVD. Furthermore, this association was robust across sensitivity analyses that accounted for the influence of each individual study.

The underlying mechanisms behind the observed association between OA and CVD risk remain unclear, but several factors may account for this relationship. First, the two diseases have some shared risk factors. Epidemiological studies have provided evidence for an association between OA and most of the traditional cardiovascular risk factors, including hypertension^19^,^20^, diabetes^21^, hypercholesterolemia^22^, and obesity^23^. Second, the most commonly prescribed drugs to relieve pain in OA patients are non-steroidal anti-inflammatory drugs (NSAIDs), and NSAIDs have been related to an increased risk of vascular events^24^. Third, OA patients are less physically active because of severe pain in the joints compared with the general population, particularly those with knee or hip OA. Physical inactivity is among the leading risk factors for CVD^25^. Finally, the most important pathological features of CVD include arterial thickening, stiffness, and atherosclerosis, which contribute to inadequate tissue perforation (ischemia). Ischemia of the bone decreases cartilage nutrition and induces multiple bone infarcts, which are characteristics of advanced OA^26^,^27^. This effect of ischemia of the bone is one potential explanation of the interrelationship between OA and CVD.

Potential limitations of this meta-analysis arise from the unavailability of individual participant data from the included studies. For instance, Jonsson *et al*.^4^ reported that the intake of polyunsaturated fatty acids was significantly more frequent in the OA group than in the control group, which potentially explained the inverse association between OA and cardiovascular events; Nielen *et al*.^12^ reported that the mean age of OA patients was significantly higher than that of controls, and thus it is likely that the OA patients with the highest risks had already died, resulting in an underrepresentation of the prevalence rate of CVD in OA patients. With more information, we would be able to analyze the associations between different types of OA and certain types of outcomes as well as better control confounding factors. To overcome this limitation, we performed subgroup analyses when possible. As a significant association was observed in most subgroups, the lack of individual participant data was not a serious limitation. The non-significant results observed in several subgroups can be partially ascribed to the 2 articles mentioned above, which did not control confounders well. Our meta-analysis was based on studies that varied in many ways (study design, population sample, adjustment for confounders, and different ascertainment methods for exposure and outcome), which may be considered another limitation. However, we adopted appropriate meta-analytic techniques with random-effect models, which enabled us to account for these differences.

The strengths of this study include the comprehensive and systematic review of the literature. Study selection, data extraction, and quality assessment were performed independently by two authors according to predesigned criteria to minimize bias and transcription errors. We included both prospective and retrospective studies that used large sample sizes, which increased the statistical power to detect potential associations. Compared to the previous meta-analysis on this topic^28^, we incorporated 3 times more articles (15 versus 5) and approximately 2 times more participants (358,944 versus 177,214) in the statistical analysis. Furthermore, the included studies had generally satisfactory designs, methods, and outcomes, and the study quality was medium to high. Finally, the consistency of the evidence overall supports a real association between OA and CVD risk.

Because OA is a very common health condition, an association between OA and CVD would be important from a public health perspective. In the general population, middle-aged people may consider screening for OA status as well as traditional cardiovascular risk factors to enable early intervention to reduce future CVD events. Patients with OA should pay more attenuation to their CVD risk. Among clinicians, cardiovascular risk must be taken into account when prescribing any non-steroidal anti-inflammatory drug for OA patients.

In conclusion, this meta-analysis provides strong evidence that OA is a significant risk factor for CVD. Given the high prevalence and incidence of OA and CVD in the general population, the observed relationship between OA and CVD has clinical and public health importance.

## Methods

### Search strategy

We carried out a meta-analysis of studies that evaluated the association between osteoarthritis and cardiovascular diseases in adults. We followed the quality of reporting of meta-analysis guidelines (the PRISMA statement) for performing and reporting the present meta-analysis (Supplementary Tables S1 and S2)^29^. Between March 2016 and May 2016, we searched Medline and EMBASE to retrieve relevant studies. The following search terms were used in different combinations: osteoarthritis, cardiovascular disease, coronary artery disease, coronary heart disease, ischemic heart disease, myocardial infarction, congestive heart failure, transient ischemic attack, and stroke. Further information was identified through a manual search of references from the extracted papers.

### Study selection

The eligibility of studies was assessed through a three-step process. First, two independent reviewers performed an initial screening of all titles and abstracts according to the following criteria: (1) original research articles were included, and other types of articles, including reviews, editors, commentaries and meta-analyses, were excluded; (2) population-based association studies reporting the relationship between OA and CVD risk were included, and articles focused on other exposure or outcomes were excluded. The full texts of all potentially relevant articles were then reviewed, and studies were included if they met the following criteria: (1) predefined diagnosis criteria for both OA and CVDs; (2) reported risk ratio (RR), hazards ratio (HR), or odds ratio (OR) estimates and 95% CIs describing the relationship between OA and risk of CVDs; (3) inclusion of a non-exposed group in prospective studies or a control group in retrospective studies. Finally, discrepancies were resolved by consensus or consultation with a third reviewer.

### Data extraction and quality assessment

We designed a standardized data collection form to extract information. Two reviewers independently performed the extraction of data. We adopted OR for retrospective studies and HR and RR for prospective studies as a measure of the association. The following characteristics were recorded in the data abstraction form: study name, authors, year of publication, residential region, type of study, source of the study population, OA and CVD definition, sample size, and adjusted confounding factors.

After the data extraction procedure, the two forms from the two reviewers were compared in a point-by-point manner, and the degree of agreement between the reviewers was assessed using kappa statistics. Any discrepancies were resolved by consensus with a third investigator while referring to the original article.

Finally, we assessed study quality according to the Newcastle–Ottawa quality assessment scale, which was recommended by the Cochrane guidelines. Details of the scoring system and the quality score of each study are listed in Supplementary Tables S3 and S4.

### Statistical analysis

For each study, we extracted the estimate and 95% confidence interval for the association result. The ORs in retrospective studies were converted to RRs for meta-analysis (RR = OR/([1 − pRef] + [pRef * OR]), where pRef is the prevalence of CVD in the control group^30^. We then performed a non-parametric sign test of the extracted estimates with a null hypothesis of “no additional increased risk of CVD in OA patients”; we considered whether studies reported a higher or lower CVD risk associated with OA, regardless of significance level or magnitude of effect.

We calculated a pooled RR estimate across all studies using a random-effects model that assumes that individual studies estimate different association effects. The random-effects model was adopted because it is probably the most conservative analysis to account for variance within and between studies.

The between-study heterogeneity was assessed by means of the *I*^2^ statistic, calculated from the Q statistic^31^. We considered the result for heterogeneity significant at P < 0.10 (two-sided) for the Q statistic. *I*^2^ > 75% was considered a high level of heterogeneity.

Publication bias was assessed by visually examining the asymmetry of a funnel plot in which the log estimates were plotted against their standard errors. Furthermore, we also employed an Egger regression test in our analysis to calculate two-tailed P values for quantifying publication bias^32^. To test the robustness of our findings, we performed a multiple-step sensitivity analysis by important study quality components and by omitting each estimate one at a time.

To explore the influence of potential sources of heterogeneity on the pooled estimate, we carried out subgroup analyses by important study characteristics, including study design (prospective or retrospective), OA sites (hand, knee or hip), OA types (radiographic or clinical), and outcomes (ischemic heart disease, stroke, congestive heart failure, or cardiovascular death). The impact of mean age and follow up time on the pooled result was explored by meta-regression. Analyses were performed with Stata Version 12.0 (StataCorp LP, College Station, TX).

## Additional Information

**How to cite this article**: Wang, H. *et al*. Osteoarthritis and the risk of cardiovascular disease: a meta-analysis of observational studies. *Sci. Rep.* **6**, 39672; doi: 10.1038/srep39672 (2016).

**Publisher's note:** Springer Nature remains neutral with regard to jurisdictional claims in published maps and institutional affiliations.

## Supplementary Material

Supplementary Information

## Figures and Tables

**Table 1:** Characteristics of the included studies.

Study	Design	Country	Cohort	CVD confirmation	Adjustment
Kadam *et al*.^9^	case-control	England and Wales	Morbidity Statistics in General Practice	medical record	Age, sex, social class, and number of other broad disease groups for which subjects consulted
Nielen *et al*.^12^	cross-sectional	Netherlands	The Netherlands Information Network of General Practice	medical record	Age, gender, hypertension and hypercholesterolemia
Jonsson *et al*.^4^	case-control	Iceland	AGES Reykjavik study	question	Age, smoking, cholesterol, triglycerides, body mass index, pulse pressure and statin use
Ong *et al*.^14^	cross-sectional	U.S.	NHANES 1999–2008	question	Age, gender, race/ethnicity, and survey period
Haara *et al*.^8^	cohort	Finland		medical record	Age, education, history of workload, smoking, and body mass index
Kishimoto *et al*.^10^	cohort	U.S.	The Honolulu Heart Program	medical record	Age, BMI, physical activity index, hypertension, diabetes mellitus, HDL cholesterol, total cholesterol, smoking status, fibrinogen, alcohol intake, and ASA and/or NSAID use
Hoeven *et al*.^6^	cohort	Netherlands	The Rotterdam Study	medical record	Age, sex, body mass index, diabetes, hypertension, total cholesterol/HDL cholesterol ratio and smoking
Tsuboi *et al*.^17^	cohort	Japan		medical record	Age, gender, BMI, and lifestyle (smoking, drinking, and exercise habits)
Barbour *et al*.^7^	cohort	U.S.	Study of Osteoporotic Fractures	medical record	Age, body mass index, education, smoking, health status, diabetes, and stroke
Haugen *et al*.^5^	cohort	U.S.	The Framingham Heart Study	medical record	Age, sex, cohort, BMI, total cholesterol: HDL ratio, current lipid-lowering treatment, increased blood pressure, current antihypertensive treatment, elevated fasting or non-fasting blood glucose, current antidiabetic treatment (oral or insulin), current use of NSAIDs, daily use of aspirin, current/previous smoking, alcohol use
Kluzek *et al*.^11^	cohort	UK	The Chingford study	medical record	Age, smoking, total cholesterol, HDL-cholesterol, systolic BP and BP medication, occupation, BMI, HRT use, past physical activity, current/previous CVD disease, non-ASA NSAIDs and glucose levels
Rahman *et al*.^16^	cross-sectional	Canada	Medical Services Plan	medical record	Age, sex, income, education, body mass index, physical activity, smoking, fruit and vegetable consumption, pain medication use, chronic obstructive pulmonary disease, hypertension and diabetes
Nuesch *et al*.^13^	cohort	England	The Somerset and Avon Survey of Health	medical record	No adjustment
Rahman *et al*.^15^	cohort	Canadian	Canadian Community Health Survey	medical record	Age, sex, family history, high cholesterol, high blood pressure, diabetes mellitus, high body mass index (BMI), smoking, and diet
Veronese *et al*.^18^	cohort	Italy	Progetto Veneto Anziani	medical record	Age; gender; waste-to-hip ratio; education level; presence at baseline of diabetes, hypertension, atrial fibrillation, chronic obstructive pulmonary disease; use at baseline of aspirin, anti-hypertensives, NSAIDSs; number of medications; smoking status; activities of daily living, mini-mental state, geriatric depression scale scores; glycosylated hemoglobin, total cholesterol, serum uric acid, estimated glomerular filtration rate, erythrocytes sedimentation rate; ankle brachial index; short physical performance battery and handgrip strength

**Table 2:** Results for subgroup analyses.

Subgroup	Included study	OA patients	CVD patients	Total participants	RR (95% CI)	P
**Study design**						
Retrospective	Refs 4,9,12,14,16	61,779	15,662	284,358	1.15 (0.95–1.38)	0.147
Prospective	Refs 5, 6, 7, 8,10,11,13,15,17,18	19,132	13,551	74,586	**1.30 (1.16–1.46)**	**<0.001**
**OA site**						
Hand	Refs 4,6,8,11,18	6,587	3,256	15,728	1.03 (0.85–1.25)	0.749
Knee	Refs 6,11,17,18	1,593	2,106	7,796	**1.30 (1.00–1.69)**	**0.047**
Hip	Refs 6,7,18	1,374	3,956	13,968	**1.23 (1.11–1.38)**	**<0.001**
**OA confirmation**						
Radiographic	Refs 4, 5, 6, 7, 8,11,13,17	8,321	5,116	25,362	**1.23 (1.02–1.48)**	**0.028**
Clinical	Refs 5,6,11	579	1,309	6,037	1.47 (0.91–2.39)	0.118
**Outcome type**						
IHD	Refs 4,5,9,10,14, 15, 16,18	71,207	8,480	177,253	**1.33 (1.20–1.46)**	**<0.001**
Stroke	Refs 4,5,14, 15, 16,18	59,429	3,469	151,321	1.11 (0.96–1.29)	0.16
CHF	Refs 5,9,14,15,18	27,516	3,389	87,500	**1.40 (1.13–1.73)**	**0.002**
Cardiovascular death	Refs 7,8,11,13,17,18	5,143	2,978	16,109	**1.53 (1.27–1.84)**	**<0.001**

## References

[b1] Mortality, G. B. D. & Causes of Death, C. Global, regional, and national age-sex specific all-cause and cause-specific mortality for 240 causes of death, 1990–2013: a systematic analysis for the Global Burden of Disease Study 2013. Lancet 385, 117–171, doi: 10.1016/S0140-6736(14)61682-2 (2015). 25530442 PMC4340604

[b2] Johnson V. L. & Hunter D. J. The epidemiology of osteoarthritis. Best practice & research. Clinical rheumatology 28, 5–15, doi: 10.1016/j.berh.2014.01.004 (2014). 24792942

[b3] Green G. A. Understanding NSAIDs: from aspirin to COX-2. Clinical cornerstone 3, 50–60 (2001). 11464731 10.1016/s1098-3597(01)90069-9

[b4] Jonsson H. . Hand osteoarthritis in older women is associated with carotid and coronary atherosclerosis: the AGES Reykjavik study. Annals of the rheumatic diseases 68, 1696–1700, doi: 10.1136/ard.2008.096289 (2009). 19033292

[b5] Haugen I. K. . Hand osteoarthritis in relation to mortality and incidence of cardiovascular disease: data from the Framingham heart study. Annals of the rheumatic diseases 74, 74–81, doi: 10.1136/annrheumdis-2013-203789 (2015). 24047870 PMC3959628

[b6] Hoeven T. A. . Disability and not osteoarthritis predicts cardiovascular disease: a prospective population-based cohort study. Annals of the rheumatic diseases 74, 752–756, doi: 10.1136/annrheumdis-2013-204388 (2015). 24385204

[b7] Barbour K. E. . Hip Osteoarthritis and the Risk of All-Cause and Disease-Specific Mortality in Older Women: A Population-Based Cohort Study. Arthritis & rheumatology 67, 1798–1805, doi: 10.1002/art.39113 (2015). 25778744 PMC4521765

[b8] Haara M. M. . Osteoarthritis of finger joints in Finns aged 30 or over: prevalence, determinants, and association with mortality. Annals of the rheumatic diseases 62, 151–158 (2003). 12525385 10.1136/ard.62.2.151 PMC1754437

[b9] Kadam U. T., Jordan K. & Croft P. R. Clinical comorbidity in patients with osteoarthritis: a case-control study of general practice consulters in England and Wales. Annals of the rheumatic diseases 63, 408–414 (2004). 15020335 10.1136/ard.2003.007526 PMC1754944

[b10] Kishimoto M. . Arthritis as a risk factor for incident coronary heart disease in elderly Japanese-American males - the Honolulu Heart Program. Bulletin of the NYU hospital for joint diseases 67, 230–235 (2009). 19583559

[b11] Kluzek S. . Painful knee but not hand osteoarthritis is an independent predictor of mortality over 23 years follow-up of a population-based cohort of middle-aged women. Annals of the rheumatic diseases, doi: 10.1136/annrheumdis-2015-208056 (2015). 26865598

[b12] Nielen M. M. . Cardiovascular disease prevalence in patients with inflammatory arthritis, diabetes mellitus and osteoarthritis: a cross-sectional study in primary care. BMC musculoskeletal disorders 13, 150, doi: 10.1186/1471-2474-13-150 (2012). 22906083 PMC3493278

[b13] Nuesch E. . All cause and disease specific mortality in patients with knee or hip osteoarthritis: population based cohort study. Bmj 342, d1165, doi: 10.1136/bmj.d1165 (2011). 21385807 PMC3050438

[b14] Ong K. L., Wu B. J., Cheung B. M., Barter P. J. & Rye K. A. Arthritis: its prevalence, risk factors, and association with cardiovascular diseases in the United States, 1999 to 2008. Annals of epidemiology 23, 80–86, doi: 10.1016/j.annepidem.2012.11.008 (2013). 23218811

[b15] Rahman M. M., Kopec J. A., Anis A. H., Cibere J. & Goldsmith C. H. Risk of cardiovascular disease in patients with osteoarthritis: a prospective longitudinal study. Arthritis care & research 65, 1951–1958, doi: 10.1002/acr.22092 (2013). 23925995

[b16] Rahman M. M., Kopec J. A., Cibere J., Goldsmith C. H. & Anis A. H. The relationship between osteoarthritis and cardiovascular disease in a population health survey: a cross-sectional study. BMJ open 3, doi: 10.1136/bmjopen-2013-002624 (2013). PMC3657665 23674445

[b17] Tsuboi M. . Do musculoskeletal degenerative diseases affect mortality and cause of death after 10 years in Japan? Journal of bone and mineral metabolism 29, 217–223, doi: 10.1007/s00774-010-0214-z (2011). 20711854

[b18] Veronese N. . Association of Osteoarthritis With Increased Risk of Cardiovascular Diseases in the Elderly: Findings From the Progetto Veneto Anziano Study Cohort. Arthritis & rheumatology 68, 1136–1144, doi: 10.1002/art.39564 (2016). 26714002

[b19] Puenpatom R. A. & Victor T. W. Increased prevalence of metabolic syndrome in individuals with osteoarthritis: an analysis of NHANES III data. Postgraduate medicine 121, 9–20, doi: 10.3810/pgm.2009.11.2073 (2009). 19940413

[b20] Conaghan P. G., Vanharanta H. & Dieppe P. A. Is progressive osteoarthritis an atheromatous vascular disease? Annals of the rheumatic diseases 64, 1539–1541, doi: 10.1136/ard.2005.039263 (2005). 16107512 PMC1755271

[b21] Louati K., Vidal C., Berenbaum F. & Sellam J. Association between diabetes mellitus and osteoarthritis: systematic literature review and meta-analysis. RMD open 1, e000077, doi: 10.1136/rmdopen-2015-000077 (2015). 26535137 PMC4613158

[b22] Hart D. J., Doyle D. V. & Spector T. D. Association between metabolic factors and knee osteoarthritis in women: the Chingford Study. The Journal of rheumatology 22, 1118–1123 (1995). 7674240

[b23] Yusuf E. . Association between weight or body mass index and hand osteoarthritis: a systematic review. Annals of the rheumatic diseases 69, 761–765, doi: 10.1136/ard.2008.106930 (2010). 19487215

[b24] Trelle S. . Cardiovascular safety of non-steroidal anti-inflammatory drugs: network meta-analysis. Bmj 342, c7086, doi: 10.1136/bmj.c7086 (2011). 21224324 PMC3019238

[b25] Ignarro L. J., Balestrieri M. L. & Napoli C. Nutrition, physical activity, and cardiovascular disease: an update. Cardiovascular research 73, 326–340, doi: 10.1016/j.cardiores.2006.06.030 (2007). 16945357

[b26] Cheras P. A., Freemont A. J. & Sikorski J. M. Intraosseous thrombosis in ischemic necrosis of bone and osteoarthritis. Osteoarthritis and cartilage/OARS, Osteoarthritis Research Society 1, 219–232 (1993). 10.1016/s1063-4584(05)80328-0 15449509

[b27] Bullough P. G. & DiCarlo E. F. Subchondral avascular necrosis: a common cause of arthritis. Annals of the rheumatic diseases 49, 412–420 (1990). 2200357 10.1136/ard.49.6.412 PMC1004114

[b28] Hall A. J., Stubbs B., Mamas M. A., Myint P. K. & Smith T. O. Association between osteoarthritis and cardiovascular disease: Systematic review and meta-analysis. European journal of preventive cardiology 23, 938–946, doi: 10.1177/2047487315610663 (2016). 26464295

[b29] Moher D., Liberati A., Tetzlaff J., Altman D. G. & Group P. Preferred reporting items for systematic reviews and meta-analyses: the PRISMA statement. PLoS medicine 6, e1000097, doi: 10.1371/journal.pmed.1000097 (2009). 19621072 PMC2707599

[b30] Zhang J. & Yu K. F. What’s the relative risk? A method of correcting the odds ratio in cohort studies of common outcomes. Jama 280, 1690–1691 (1998). 9832001 10.1001/jama.280.19.1690

[b31] Higgins J. P. & Thompson S. G. Quantifying heterogeneity in a meta-analysis. Statistics in medicine 21, 1539–1558, doi: 10.1002/sim.1186 (2002). 12111919

[b32] Egger M., Davey Smith G., Schneider M. & Minder C. Bias in meta-analysis detected by a simple, graphical test. Bmj 315, 629–634 (1997). 9310563 10.1136/bmj.315.7109.629 PMC2127453

