# Supplementary material for: Osteoarthritis and the risk of cardiovascular disease: a meta-analysis of observational studies
Source: Sci Rep. 2016 Dec 22;6:39672. doi: 10.1038/srep39672 (PMC5177921; doi:10.1038/srep39672)
Supplement: Supplementary Information [file srep39672-s1.pdf]

Osteoarthritis and the risk of cardiovascular disease: a meta-analysis of observational studies

Haoran Wang[1*], Jing Bai[2], Bing He[3], Xinrong Hu[1], Dongliang Liu[1*]

[1]Department of Cardiology, [2]Department of Endocrinology and Diabetes, [3]Department of Joint Surgery, Luohe Central Hospital, The First Affiliated Hospital of Luohe Medical College, Luohe 462000, China

*Corresponding Author: Haoran Wang or Dongliang Liu

Department of Cardiology

Luohe Central Hospital, The First Affiliated Hospital of Luohe Medical College

56# Renmin Ave.,

Luohe 462000, People's Rep. of China

Tel. & Fax: 86-395-3356-758

Email: washingtonhr@163.com or dongliang003@live.cn

Haoran Wang and Jing Bai contributed equally to this work.

Supplementary file

Contents:

Table S1. PRISMA 2009 Checklist.

Table S2. PRISMA 2009 Flow Diagram.

Table S3. Quality score assessment criteria for retrospective studies.

Table S4. Quality score assessment criteria for prospective studies.

Figure S1. Funnel plots for bias assessment.

Figure S2. Sensitivity analyses by omitting one study at a time.

Figure S3. Meta-regression analysis investigating potential effect of the follow-up time on CVD risk associated with OA.

Figure S4. Meta-regression analysis investigating potential effect of the mean age of the exposed group on CVD risk associated with OA.

Figure S5. Meta-regression analysis investigating potential effect of the mean age of the non-exposed group on CVD risk associated with OA.

Table S1-PRISMA 2009 Checklist

Section/topic	#	Checklist item	Reported on page #
**TITLE**			
Title	1	Identify the report as a systematic review, meta-analysis, or both.	1
**ABSTRACT**			
Structured summary	2	Provide a structured summary including, as applicable: background; objectives; data sources; study eligibility criteria, participants, and interventions; study appraisal and synthesis methods; results; limitations; conclusions and implications of key findings; systematic review registration number.	2
**INTRODUCTION**			
Rationale	3	Describe the rationale for the review in the context of what is already known.	3
Objectives	4	Provide an explicit statement of questions being addressed with reference to participants, interventions, comparisons, outcomes, and study design (PICOS).	3, 4
**METHODS**			
Protocol and registration	5	Indicate if a review protocol exists, if and where it can be accessed (e.g., Web address), and, if available, provide registration information including registration number.	NA
Eligibility criteria	6	Specify study characteristics (e.g., PICOS, length of follow-up) and report characteristics (e.g., years considered, language, publication status) used as criteria for eligibility, giving rationale.	14, 15
Information sources	7	Describe all information sources (e.g., databases with dates of coverage, contact with study authors to identify additional studies) in the search and date last searched.	14
Search	8	Present full electronic search strategy for at least one database, including any limits used, such that it could be repeated.	14
Study selection	9	State the process for selecting studies (i.e., screening, eligibility, included in systematic review, and, if applicable, included in the meta-analysis).	14, 15
Data collection process	10	Describe method of data extraction from reports (e.g., piloted forms, independently, in duplicate) and any processes for obtaining and confirming data from investigators.	15
Data items	11	List and define all variables for which data were sought (e.g., PICOS, funding sources) and any assumptions and simplifications made.	15
Risk of bias in individual studies	12	Describe methods used for assessing risk of bias of individual studies (including specification of whether this was done at the study or outcome level), and how this information is to be used in any data synthesis.	15, 16
Summary measures	13	State the principal summary measures (e.g., risk ratio, difference in means).	16
Synthesis of results	14	Describe the methods of handling data and combining results of studies, if done, including measures of consistency (e.g., I^2) for each meta-analysis.	16, 17

Table S1-PRISMA 2009 Checklist

Section/topic	#	Checklist item	Reported on page #
Risk of bias across studies	15	Specify any assessment of risk of bias that may affect the cumulative evidence (e.g., publication bias, selective reporting within studies).	16, 17
Additional analyses	16	Describe methods of additional analyses (e.g., sensitivity or subgroup analyses, meta-regression), if done, indicating which were pre-specified.	16, 17
**RESULTS**			
Study selection	17	Give numbers of studies screened, assessed for eligibility, and included in the review, with reasons for exclusions at each stage, ideally with a flow diagram.	5
Study characteristics	18	For each study, present characteristics for which data were extracted (e.g., study size, PICOS, follow-up period) and provide the citations.	5, 6
Risk of bias within studies	19	Present data on risk of bias of each study and, if available, any outcome level assessment (see item 12).	6
Results of individual studies	20	For all outcomes considered (benefits or harms), present, for each study: (a) simple summary data for each intervention group (b) effect estimates and confidence intervals, ideally with a forest plot.	6, 7
Synthesis of results	21	Present results of each meta-analysis done, including confidence intervals and measures of consistency.	6-9
Risk of bias across studies	22	Present results of any assessment of risk of bias across studies (see Item 15).	7
Additional analysis	23	Give results of additional analyses, if done (e.g., sensitivity or subgroup analyses, meta-regression [see Item 16]).	7-9
**DISCUSSION**			
Summary of evidence	24	Summarize the main findings including the strength of evidence for each main outcome; consider their relevance to key groups (e.g., healthcare providers, users, and policy makers).	10-13
Limitations	25	Discuss limitations at study and outcome level (e.g., risk of bias), and at review-level (e.g., incomplete retrieval of identified research, reporting bias).	11, 12
Conclusions	26	Provide a general interpretation of the results in the context of other evidence, and implications for future research.	13
**FUNDING**			
Funding	27	Describe sources of funding for the systematic review and other support (e.g., supply of data); role of funders for the systematic review.	21

From: Moher D, Liberati A, Tetzlaff J, Altman DG, The PRISMA Group (2009). Preferred Reporting Items for Systematic Reviews and Meta-Analyses: The PRISMA Statement. PLoS Med 6(6): e1000097. doi:10.1371/journal.pmed1000097

For more information, visit: www.prisma-statement.org.

Table S2-PRISMA 2009 Flow Diagram

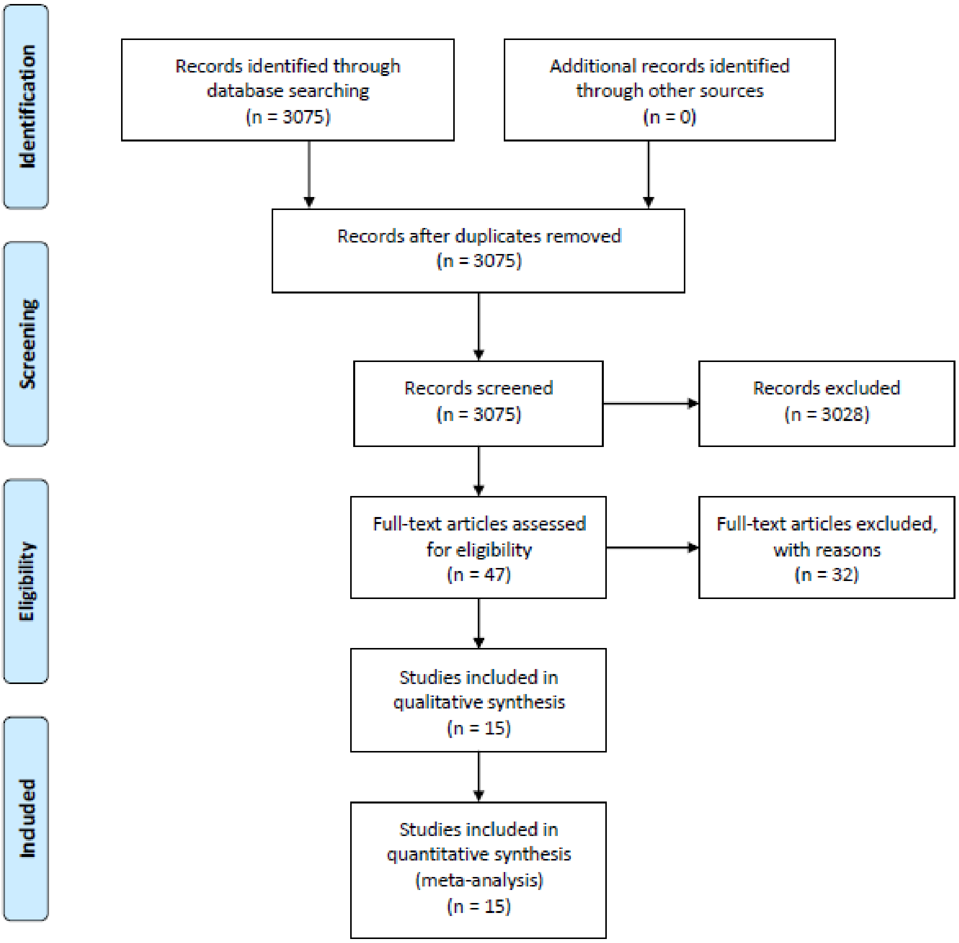

From: Moher D, Liberati A, Tetzlaff J, Altman DG, The PRISMA Group (2009). *P*referred *R*eporting *I*tems for *S*ystematic Reviews and *M*eta-*A*nalyses: The PRISMA Statement. PLoS Med 6(6): e1000097. doi:10.1371/journal.pmed1000097

For more information, visit www.prisma-statement.org.

Table S3. Quality score assessment criteria for retrospective studies.

**Selection**	Score	13*	16*	18*	20*	4*
1) Is the case definition adequate?						
a) yes, with independent validation	2					
b) yes, eg record linkage or based on self reports	1	1	1	1	1	1
c) no description	0					
2) Representativeness of the cases						
a) consecutive or obviously representative series of cases	1	1	1	1	1	1
b) potential for selection biases or not stated	0					
3) Selection of Controls						
a) community controls	2	2	2	2	2	2
b) hospital controls	1					
c) no description	0					
4) Definition of Controls						
a) no history of disease (endpoint)	1	1	1	1	1	1
b) no description of source	0					
**Comparability**						
1) Comparability of cases and controls on the basis of the design or analysis						
a) study controls for age	2	2	2	2	2	2
b) study controls for any additional factor	1					
c) no adjustment	0					
**Exposure**						
1) Ascertainment of exposure						
c) clinical/radiographic	2					2
d) medical record	1	1	1	1	1	
e) written self report	0					
2) Same method of ascertainment for cases and controls						
a) yes	1	1	1	1	1	1
b) no	0					
Total score	11	9	9	9	9	10

*Number indicates the cited article.

Quality level was defined as low (≤5), medium (6-8) or high (≥9) according to quality score.

Table S4. Quality score assessment criteria for prospective studies.

**Selection**	Score	22*	12*	14*	6*	21*	11*	5*	15*	17*	19*
1) Representativeness of the exposed cohort											
a) truly representative of the average OA patients in the community	2	2	2	2	2	2	2	2	2	2	2
b) somewhat representative of the average OA patients in the community	1										
c) selected group of users eg nurses, volunteers	0										
d) no description of the derivation of the cohort	0										
2) Selection of the non exposed cohort											
a) drawn from the same community as the exposed cohort	2	2	2	2	2	2	2	2	2	2	2
b) drawn from a different source	1										
c) no description of the derivation of the non exposed cohort	0										
3) Ascertainment of exposure											
c) clinical/radiographic	2	2	2		2	2	2	2	2	2	
d) medical record	1										1
e) written self report	0			0							
4) Demonstration that outcome of interest was not present at start of study											
a) yes	1	1	1	1	1	1	1	1	1	1	1
b) no	0										
**Comparability**											
1) Comparability of cohorts on the basis of the design or analysis											
a) study controls for age	2	2	2	2	2	2	2	2	2		2
b) study controls for any additional factor	1										
c) no adjustment	0									0	
**Outcome**											
1) Assessment of outcome											
a) independent blind assessment	3				3			3	3		
b) record linkage	2	2	2	2		2	2			2	2
c) self report	1										
d) no description	0										
2) Was follow-up long enough for outcomes to occur											
a) yes (select an adequate follow up period for outcome of interest)	1	1	1	1	1	1	1	1	1	1	1
b) no	0										
Total score	13	12	12	10	13	12	12	13	13	10	11

*Number indicates the cited article.

Quality level was defined as low (≤7), medium (8-10) or high (≥11) according to quality score.

Supplementary figure legends

Figure S1. Funnel plots for bias assessment. Each plot represents a separate study estimate. Egger's test, P = 0.659.

Figure S2. Sensitivity analyses by omitting one study at a time. The summary RR (95% CI) was indicated by each horizontal line when the labeled study was omitted and the reminders were reanalyzed. And the omission of any one study did not appreciably change the pooled RR.

Figure S3. Meta-regression analysis investigating potential effect of the follow-up time on CVD risk associated with OA.

Circles represent individual results with the size of the circle being proportional to its weight in the random-effects meta-analysis. Meta-regression line (solid line) was estimated using a random-effect linear meta-regression model with follow-up time as the covariate. The result indicated that there was no significant effect (p = 0.308) of follow-up time on CVD risk associated with OA.

Figure S4. Meta-regression analysis investigating potential effect of the mean age of the exposed group on CVD risk associated with OA. Circles represent individual results with the size of the circle being proportional to its weight in the random-effects meta-analysis. Meta-regression line (solid line) was estimated using a random-effect linear meta-regression model with mean age of OA

group (the exposed group) as the covariate. The result indicated that there was no significant effect ($p = 0.196$) of mean age of the exposed group on CVD risk associated with OA.

Figure S5. Meta-regression analysis investigating potential effect of the mean age of the non-exposed group on CVD risk associated with OA.

Circles represent individual results with the size of the circle being proportional to its weight in the random-effects meta-analysis. Meta-regression line (solid line) was estimated using a random-effect linear meta-regression model with mean age of control group (the non-exposed group) as the covariate. The result indicated that there was no significant effect ($p = 0.575$) of mean age of the non-exposed group on CVD risk associated with OA.

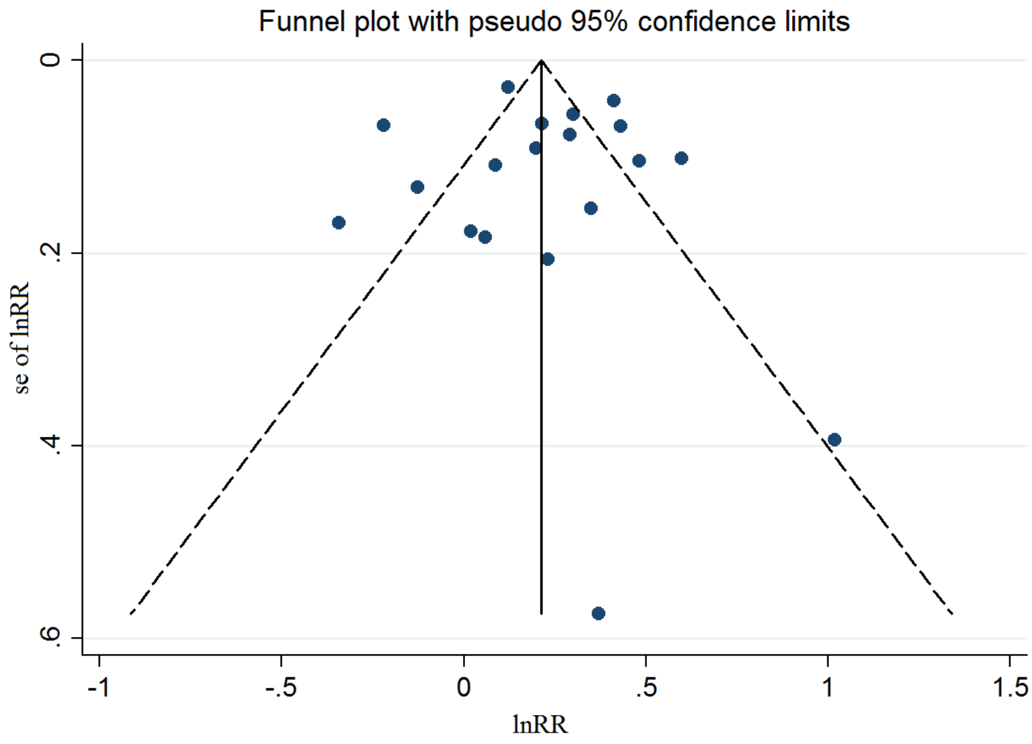

Figure S1. Funnel plots for bias assessment.

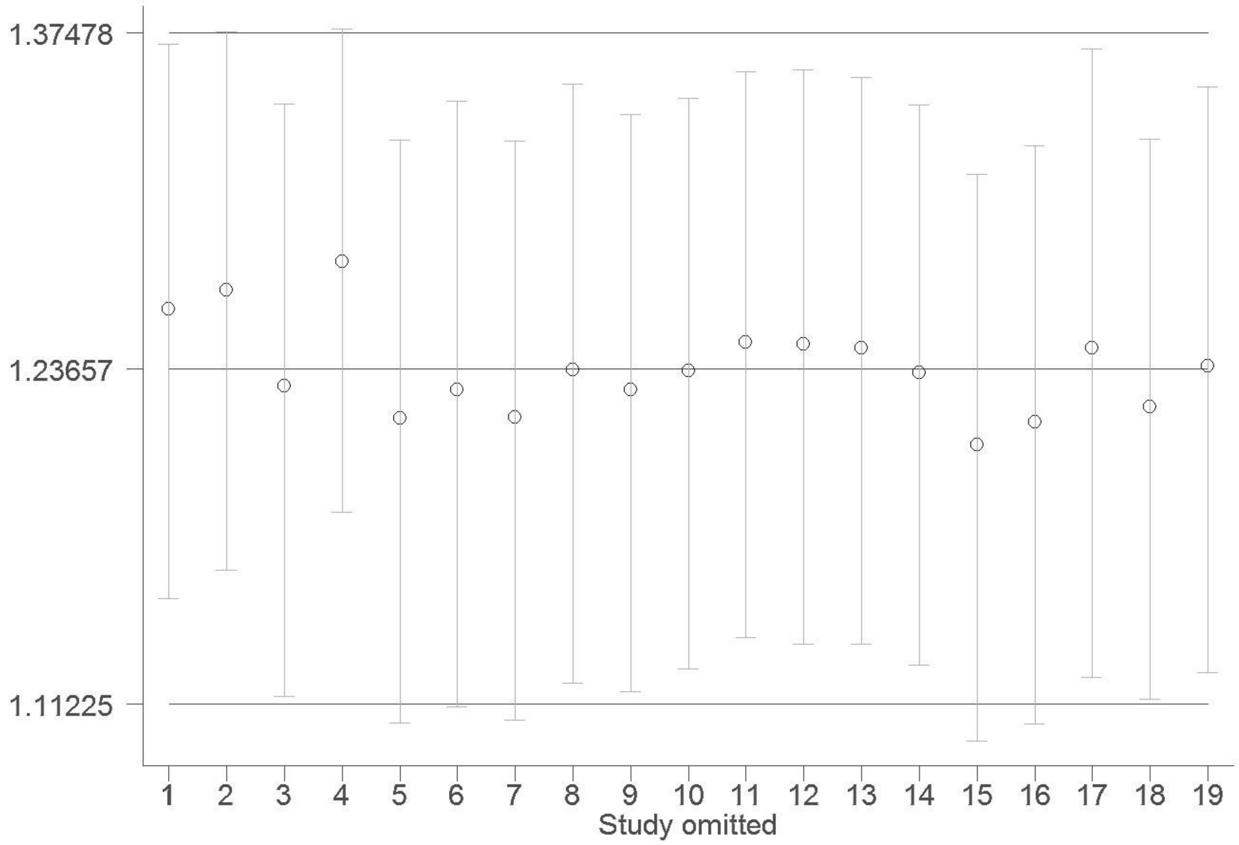

Figure S2. Sensitivity analyses by omitting one study at a time.

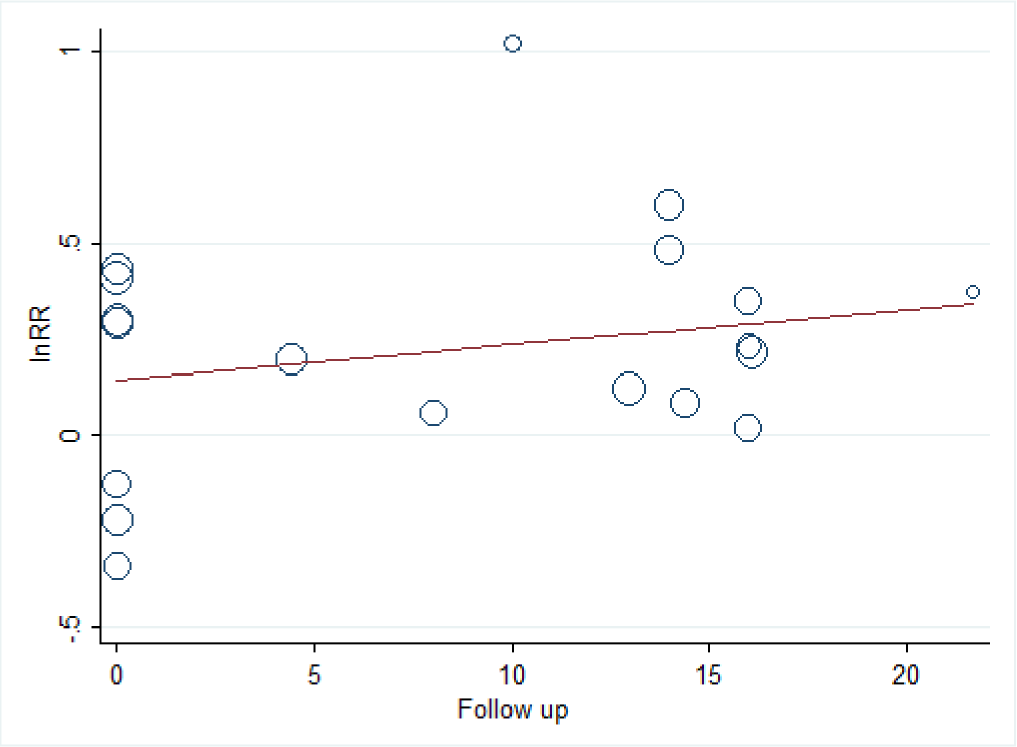

Figure S3. Meta-regression analysis investigating potential effect of the follow-up time on CVD risk associated with OA.

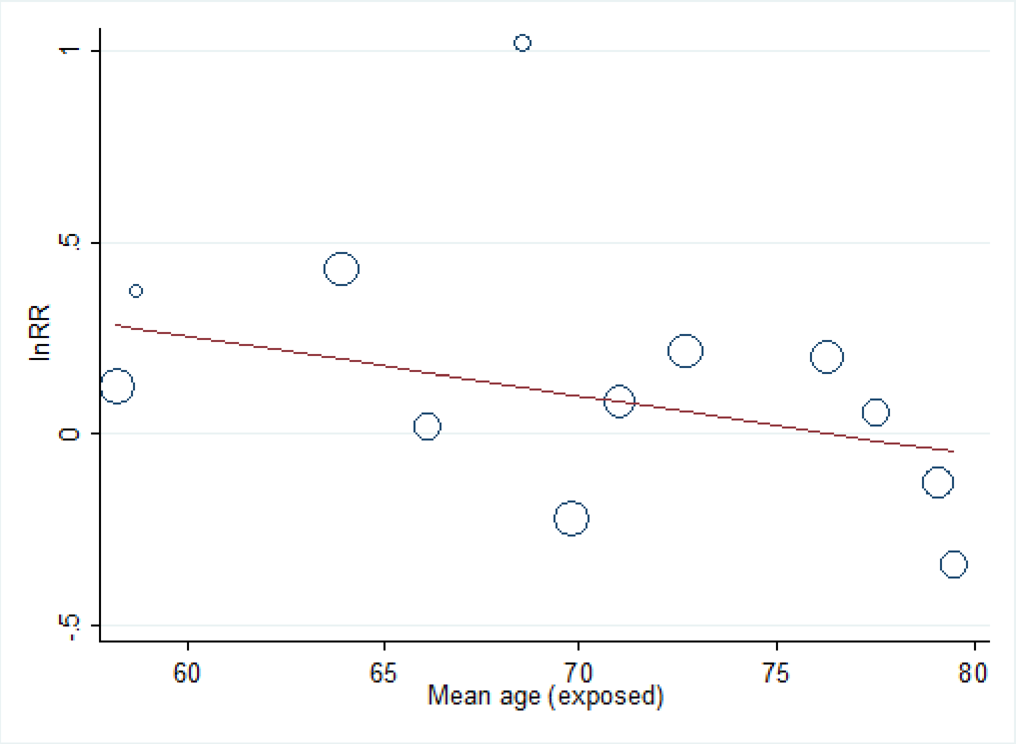

Figure S4. Meta-regression analysis investigating potential effect of the mean age of the exposed group on CVD risk associated with OA.

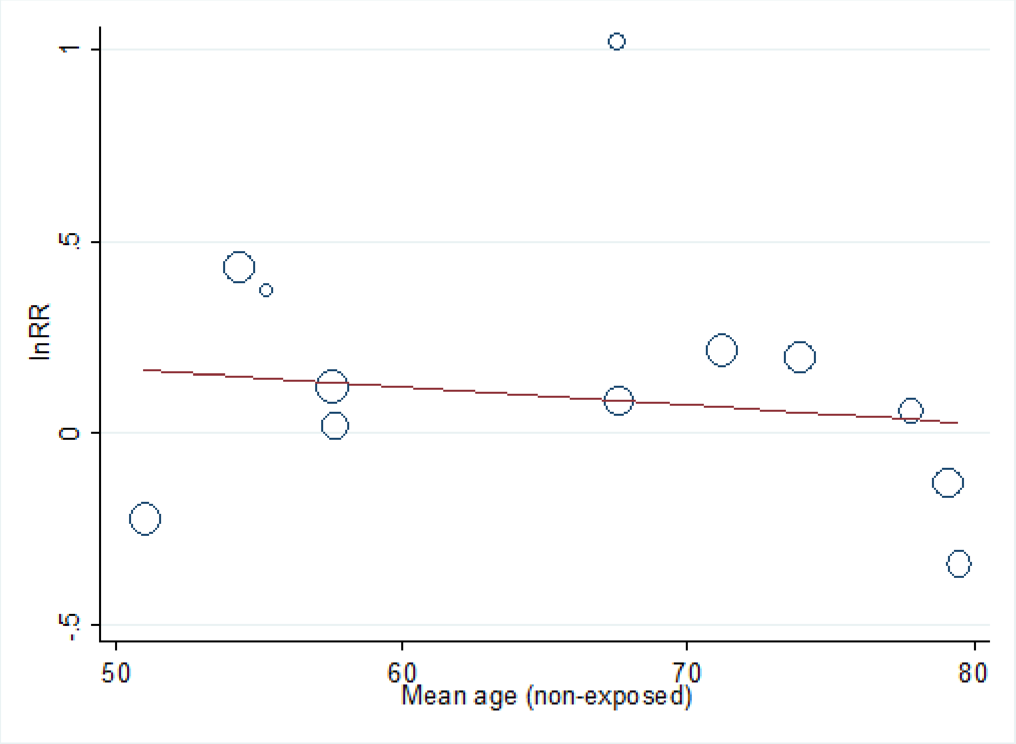

Figure S5. Meta-regression analysis investigating potential effect of the mean age of the non-exposed group on CVD risk associated with OA.
